# Antiproliferative Effects of the Methanolic Petiole Extract of Eichhornia crassipes Against Sloan Kettering Melanoma 5 Cell Line: An In Vitro Study

**DOI:** 10.7759/cureus.30554

**Published:** 2022-10-21

**Authors:** Noufal K P, B Rajesh, Sujith S Nair

**Affiliations:** 1 Department of Anatomy, Bharath Institute of Higher Education and Research, Chennai, IND; 2 Department of Anatomy, Sri Lakshmi Narayana Institute of Medical Sciences, Pondicherry, IND; 3 Department of Pharmaceutics, Crescent College of Pharmaceutical Sciences, Kannur, IND

**Keywords:** mtt assay, probit analysis, methanolic extract, eichhornia crassipes, cell growth inhibition

## Abstract

Background

*Eichhornia crassipes* (*E. crassipes*) have several secondary metabolites that have medicinal value. These include sterols, alkaloids, phenolics, flavonoids, tannins, and saponins. In the current study, the methanolic petiole extract of *E. crassipes* was examined to determine its potential antiproliferative activity against Sloan Kettering Melanoma 5 (SK-Mel-5) cell lines.

Materials and methods

*Eichhornia* *crassipes* were obtained from the water bodies of Ezhikkara, Ernakulam, Kerala. The Soxhlet technique was used to produce the extract. Leaves, petioles, and roots were dried and pulverized before being analyzed phytochemically in a number of solvents. The 3-(4,5-dimethylthiazol-2-yl)-2,5-diphenyltetrazolium bromide (MTT) assay was used to measure the extent to which various dosages of the extract inhibited cell proliferation, and the methanolic extract of petioles was chosen since it contained more anticancer components. The half maximal inhibitory concentration (IC_50_) was determined by utilizing a probit model and the slope-gradient method of the regression equation. The Statistical Package for Social Sciences (SPSS) version 21 (IBM SPSS Statistics, Armonk, NY) was used for the analysis.

Results

We examined the effects of 12.5, 25, 50, 100, and 200 μg/ml of methanolic petiole extract. The data indicated that the methanol extract significantly reduced SK-Mel-5 cell viability. Cell growth inhibition increased with concentration but was shown to be relatively low at 100 g/ml, exhibiting 38.911% of inhibitory activity. The percentage of cell growth inhibition at 200 g/ml was 52.965%. The methanolic petiole extracts of *E. crassipes* were found to be cytotoxic with IC_50_ values of 172.186 g/ml. Probit analysis was performed to obtain the regression equation.

Conclusion

The in vitro study suggests that the methanol extract of the petiole of *E. crassipes* had modest antiproliferative action against SK-Mel-5 cells, a typical human melanocyte tumor cell line. The study findings shed light on the anticancer activity of *E. crassipes*, making it an appropriate source of drug-lead chemicals for the development of safer and cost-effective remedies for cutaneous ailments varying from rashes to awful melanoma.

## Introduction

Plants are employed for therapeutic purposes in several countries and are the source of many robust and powerful medications. Secondary metabolites have been used to identify the active ingredients of several medications derived from plants. Only 10% of around 12,000 bioactive phytomolecules have been chemically reported. Alkaloids, phenolics, flavonoids, polyphenols, and essential oils were identified as medicinally beneficial bioactive ingredients. Alkaloids perform a vital metabolic function in biological systems and regulate development [1,2]. Pharmaceuticals from plant species constitute 25% of overall drugs in industrialized nations and nearly 80% in underdeveloped countries [3]. The strategy of the World Health Organization is to advocate, promote, and enable the use of competent medicinal herbs in developing nations for various health programs [4].

Cutaneous melanoma presents a challenging medical intervention owing to its exceedingly invasive nature. It has a greater potential to produce metastases, leading to decreased survival and increased mortality rates. The current existing drugs, such as aldesleukin, binimetinib, and Braftovi (encorafenib), available on the market to treat individuals with melanoma have numerous adverse effects, in addition to being ineffective in deterring the deadly progression of the disease. Various studies on innovative treatment techniques have been attempted to overcome the high resistance of melanoma to standard chemotherapy [5]. Plants are an infinite source of pharmacologically active natural compounds with promising prospects for treating tumors while posing relatively minimal toxicity [6]. Plant products have been found to have medical efficacy due to the inclusion of bioactive components such as alkaloids, tannins, flavonoids, and phenolic compounds.

Water hyacinth, or *Eichhornia crassipes*, is a perennial plant native to South America that floats on the water's surface. This plant's native range is Brazil, although it has been successfully transplanted to various tropical and subtropical regions. The phytomolecules found in *E. crassipes* are packed with powerful antioxidant activity [7,8]. This class includes phenolic acids, sterols, terpenoids, and many more. L-Galactose, L-arabinose, and D-xylose are only some of the many primary metabolites that have been extracted from the plant; others include hemicellulose, cellulose, glycolipids, and triacylglycerols. The primary bioactive metabolites were found to be accompanied by a number of secondary bioactive metabolites (10.49%). Flavonoids accounted for 10%, fatty acids for 10%, polyphenols for 9.73%, alkaloids for 7.2%, sterols for 6.17%, and other compounds for 19.13%. Floral parts, leaf surfaces, stems, and roots all contain phospholipids, with phosphatidylethanolamine, phosphatidylcholine, and phosphatidylglycerol being the most frequent. Simple phenols were isolated in extracts of leaves, petioles, and flowers of *E. crassipes* retrieved from India [8].

*Eichhornia crassipes* is one of the most noxious aquatic weeds on the planet. Many people believe that this plant contaminates water, but in fact, it is a gift of nature that provides a wide range of components that may aid in the treatment of various ailments [8]. It can be employed to make animal feed, handicrafts such as bags and accessories, organic fertilizers, biofuel, and biogas generation [9]. This macrophyte possesses two distinct morphologies with intermediaries that change depending on the environment in which it thrives. In one of the morphological types, the petioles are elongated up to approximately 1 m in nutrient-enriched waterways free of herbivores and have circular leaves in dense groups. Leaves of the second morphological kind are kidney-shaped, and their petioles are less than 30 cm long and bulbous in areas where the plants are not clumped together in dense mats or on the edge of infestations [9].

Oxygen free radicals are released as a result of stress-related disorders and are reported to be associated with organ failure and death. The antioxidants greatly reduce free radicals and mitigate cellular damage. The 2,2-diphenyl-1-picrylhydrazyl (DPPH) and ferric-reducing ability of plasma (FRAP) tests were used to identify antioxidants in plant life. In both tests, methanol extracts performed better than ethanol extracts. Plant extract chemicals contain antibacterial, antiviral, antialgal, antioxidant, anti-inflammatory, cytotoxic, and cardioprotective effects, and they may also inhibit the production of reactive oxygen species and free radicals [10,11].

The root, stem, and leaf portions of the plant had the highest alkali solubility values of 54%, 52%, and 51%, respectively [12]. Due to its useful properties, *E. crassipes* has been the focus of harvesting efforts across the globe to be used as a commercial source. Despite this, the only use of the herb is at the folkloric level, and there have been few documented medicinal studies on it [1]. The current study was conducted on methanolic petiole extract of *E. crassipes* to determine its potential antiproliferative activity against Sloan Kettering Melanoma 5 (SK-Mel-5) cell lines.

## Materials and methods

Plant collection and identification

From the waters of Ezhikkara, Ernakulam, Kerala, we were able to collect *E. crassipes*. The University of Calicut's Department of Botany experts validated the plant's identity [3]. The study was ethically approved by Sri Lakshmi Narayana Institute of Medical Sciences with ethical number IEC/C-P/08/2020. Ezhikkara is a traditional village in the Paravur block of Panchayat that is surrounded by lagoons and lush vegetation. It is located between latitude 10° 8' 25.7856" North and longitude 76° 13' 49.8792" East. The blooming of water hyacinths in these bodies of water wreaks havoc on subsistence farmers by significantly reducing water flow and depleting oxygen in aquaculture farms. The fish raised in cages are the most vulnerable. It also serves as a breeding site for parasites and other harmful species that cause fish ailments. The government has initiated awareness programs about the negative impacts of water hyacinths on the aquaculture industry and measures to eradicate them from aquatic bodies. Consequently, an attempt was made to evaluate its anticancer activity in vitro against malignant cells.

Preparation of an Extract

The entire plant was identified and removed from the lake. The aerial portions were properly cleaned multiple times under running water. It was then thoroughly cleaned with distilled and sterilized water. The fresh leaves, petioles, and roots were isolated separately and chopped up into tiny pieces before being air-dried or lyophilized. After being ground into a fine powder, it was fractionated in a Soxhlet device with methanol, ethanol, chloroform, acetone, and water, which also helped in dehydration. Solvents were recovered from the separated samples by distilling them in a rotary evaporator at a temperature of 40°C. Low pressure was used to concentrate the extracts until they were completely dry. The extracted fractionates were stored in the refrigerator for future use.

Phytochemical analysis

Phytoconstituents such as alkaloids, flavonoids, terpenoids, steroids, phlobatannins, and saponins were identified in extracts of leaves, petioles, and roots using standard techniques such as ultraviolet (UV)-visible spectroscopy, Infrared (IR), nuclear magnetic resonance (NMR), and mass spectroscopy. Based on the phytochemistry, the methanolic extract of petioles was chosen for the 3-(4,5-dimethylthiazol-2-yl)-2,5-diphenyltetrazolium bromide (MTT) test because it contained a higher concentration of anticancer components.

In vitro evaluation of anticancer activity by MTT assay cell culture

The SK-Mel-5 human malignant melanoma cell line was obtained from the National Centre for Cell Science (NCCS), Pune, India.

Cell culture media and maintenance

Dulbecco's Modified Eagle's Medium (DMEM) with 10% inactivated fetal bovine serum (FBS) and 1% of a mixture of penicillin (100 U/ml), streptomycin (100 g/ml), and amphotericin B (2.5 g/ml) was used for the cells' growth and refinement. The cells were grown in a tissue culture (TC) incubator (Galaxy® 170, Eppendorf, Hamburg, Germany) at 37°C in a humid environment with a carbon dioxide limit of 5%.

Cell preservation

The cells were stored at low passage numbers in the liquid nitrogen vapor phase using modified cell culture media enriched with 20% FBS and 10% dimethylsulfoxide (DMSO) or glycerol.

In vitro screening of cytotoxicity

Trypsinization was performed on 80%-90% of adherent cells cultured in tissue culture (TC) flasks. Trypsinization is the technique of removing confluent cells from a TC flask in order to subculture them or seed 96-well plates for assays. We next exposed the cells that had been cultured in a TC cup to a solution containing 0.025% trypsin and 0.01% ethylenediaminetetraacetic acid (EDTA) in phosphate-buffered saline. Cells were trypsinized and then seeded at a density of 5000 cells per well in a total volume of 100 L. The 96-well plates were stored in a cell culture incubator for three to four days.

Sample preparation and treatment

To guarantee sterility, samples in DMEM (100 mg/ml) were filtered via a 0.2 m Millipore syringe filter. At 12.5, 25, 50, 100, and 200 g, the material was diluted in DMEM fluid before being implanted into 96-well plates containing grown cells. Nonexperimental wells served as controls. In order to minimize the possibility of mistakes, we ran each experiment three times and took the average. After the test samples had been applied to the plates, they were put into an incubator for 24 hours.

Direct microscopic observation

Before, during, and after a 24-hour incubation period, pictures of the treatment and control wells were taken with an inverted phase contrast tissue culture microscope (Labomed TCM-400 {Labomed Inc., Los Angeles, CA} with MICAPSTM HD camera).

The Principle and Procedure of MTT Assay

The MTT test evaluates metabolic activity inside cells to determine cell viability, proliferation, and cytotoxicity. The formation of purple formazan crystals from a yellow tetrazolium or MTT salt is the basis of this colorimetric test. Cells in culture convert MTT to formazan via nicotinamide adenine dinucleotide phosphate hydrogen (NAD(P)H)-dependent oxidoreductase enzymes [13]. An enzyme-linked immunosorbent assay (ELISA) plate reader calibrated to detect absorbance at 570 nm is used to determine the concentration of the purple solution produced after dissolving the insoluble formazan crystals in 100% DMSO.

The material from the wells was aspirated and disposed of after sample treatment and incubation of 24 hours. A DMEM with 0.5 mg/ml MTT solution was used to fill each well to a final volume of 100 L. Two to four hours later, the plates were put into an incubator to encourage the growth of formazan crystals. We discarded the supernatant in its whole and added 100 µl of 100% DMSO to each well. Two wells per plate were left empty to serve as controls. With a microplate reader, the optical density of the absorbance at 570 nm was evaluated and compared to that of the control. The average of the three sets of measurements was used to figure out what percentage of cells were still alive: percentage of cell viability = (average absorbance of treated/average absorbance of control) × 100.

SPSS (IBM SPSS Statistics, Armonk, NY) software was used to do the statistical analysis. The percentages of cell viability and cell growth inhibition were computed from the absorbance measurements. Toxic or dangerous substances may be measured by their IC_50_, which is defined as the concentration required to reduce biological processes by 50%. The IC_50_ was found using probit analysis, and the value was calculated using the slope-gradient of the equation y = mx + c.

## Results

The cytotoxicity of *E. crassipes's* petiole methanol extract was tested on the SK-Mel-5 cell line (Table 1).

**Table 1:** Probit analysis of the methanolic extract of Eichhornia crassipes against SK-Mel-5 cell lines SD: standard deviation; µ: micro; SK-Mel-5: Sloan Kettering Melanoma 5; abs: absorbance

			Drug concentration unit: µg/ml (cell line: SK-Mel-5)				
Parameter	Blank	Untreated	12.5 µg/ml	25 µg/ml	50 µg/ml	100 µg/ml	200 µg/ml
Abs reading 1	0.057	1.273	1.189	1.069	0.947	0.804	0.643
Abs reading 2	0.045	1.296	1.177	1.076	0.951	0.806	0.626
Abs reading 3	0.053	1.279	1.186	1.063	0.947	0.803	0.625
Mean abs ± SD	0.052 ± 0.006	1.283 ± 0.012	1.184 ± 0.006	1.069 ± 0.007	0.948 ± 0.002	0.804 ± 0.002	0.631 ± 0.01
Mean abs (sample blank)	0	1.231	1.132	1.017	0.896	0.752	0.579
Cell viability (%)	0	100	91.958	82.616	72.786	61.089	47.035
Cell growth inhibition (%)	0	0	8.042	17.384	27.214	38.911	52.965

**Figure 1:**
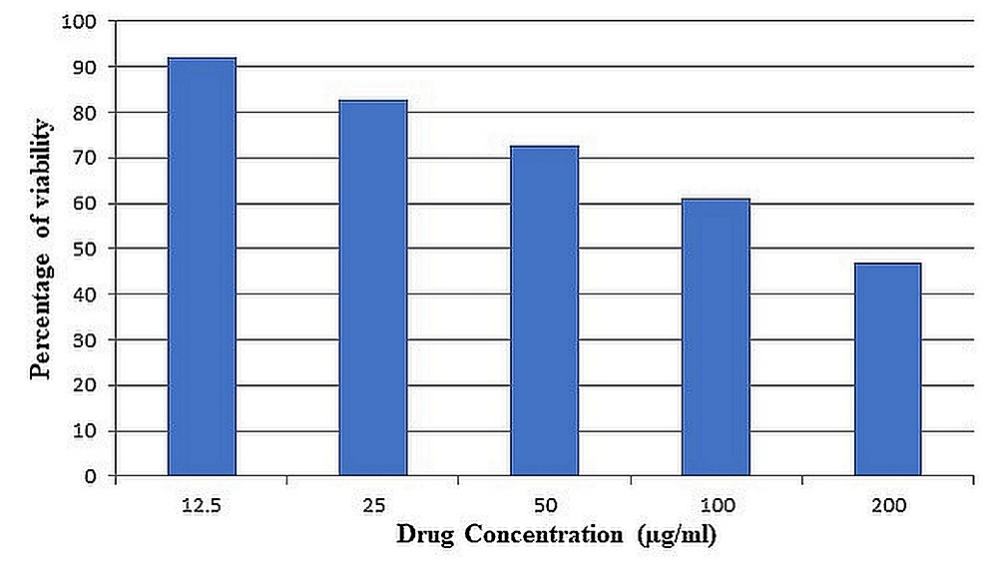
Anticancer activity of the methanolic extract of Eichhornia crassipes against SK-Mel-5 cell line µ: micro; SK-Mel-5: Sloan Kettering Melanoma 5

According to the results of the investigation, the tumor cell lines' viability was significantly reduced by the methanol extract. Extracts prepared in methanol were tested at doses of 12.5, 25, 50, 100, and 200 g/ml, with the highest showing the most efficacy. The percentage of cell growth that could be inhibited was shown to rise with concentration, albeit it was still somewhat low at 100 g/ml, at 38.911%. Cell proliferation was suppressed by 52.96% at 200 g/ml. Figure 1 depicts the impact of different doses of *E. crassipes* extracts on the growth of SK-Mel-5 cell lines.

**Figure 2:**
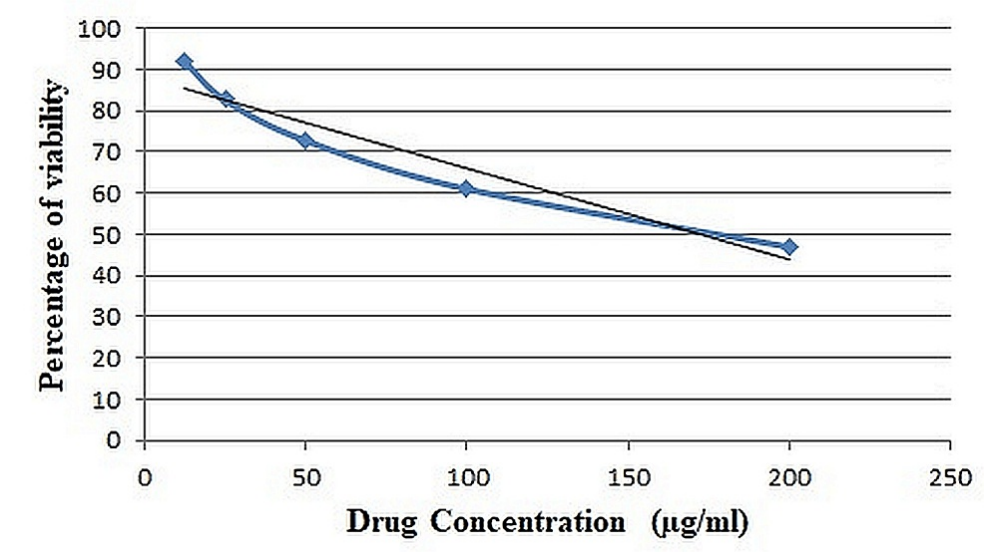
Dose response curve of the methanolic extract of Eichhornia crassipes against SK-Mel-5 cell line SK-Mel-5: Sloan Kettering Melanoma 5

The methanolic extract of petioles of *E. crassipes* demonstrated a greater cytotoxic effect on the cell lines than the control group, based on the study findings. It can be seen in Figure 2 that when the concentration of the extract was raised, the percentage of viable cells and the number of proliferating cells in each cell line rose dramatically.

There was a dose-dependent decrease in SK-Mel-5 cell viability after treatment with a methanolic extract of petiole of *E. crassipes*. In IC_50_ values of 172.186 g/ml of the test sample concentration, methanolic extracts of *E. crassipes* were shown to be cytotoxic against the tumor cell lines. Probit analysis was performed to obtain the regression equation y = −0.2228 × +88.363 and R2 = 0.9233.

## Discussion

Water hyacinth, one of the most prolific plants, is also a huge environmental threat due to its invasive nature as an aquatic weed. Water hyacinth is a problem due to its negative effects on the environment and the economy, yet the commercial use of the plant may be one solution for managing the problem [14]. Depending on factors such as plant genotype, seasonal shifts, and geographic location, the physiochemical components of aquatic weeds and plants may vary greatly [12]. The present study's phytochemical analysis of the plant showed that the petiole sections of *E. crassipes* had the highest amounts of anticancer components.

In accordance with projections from the International Agency for Research on Cancer (IARC), the annual number of new cancer cases is projected to increase by more than two-thirds by the year 2030. Cancer's supposedly heavy toll means it inhibits progress in many ways, economically and otherwise [15]. Phytochemicals reduce colon cancer risk and stop tumor spread in animal studies [16]. *Eichhornia crassipes* leaves have a cytotoxic potency of 44% against the lung cancer gene-alteration profile of human lung cancer cell lines (NCI-H322 cell line) and 20%-31% against the breast cancer T47D cell line [17]. Tyrosinase inhibitors have been shown to have antiproliferative effects against melanoma cell lines. The inhibitory effects of the plant extracts discovered in the current study against the melanotic SK-Mel-5 cell line can be attributed, at least partially, to the tyrosinase inhibitory activity, which was consistent with a similar study by Fraihat et al. [18].

In comparison to conventional procedures employed for cytotoxicity assays, the colorimetric MTT assay utilized in the current work is a versatile and quantitative method. The assay uses only three reagents: MTT, isopropanol, and hydrochloric acid. They were scanned by a plate reader with a high scanning rate of 90 seconds per 96 wells [13]. The results of the present investigation demonstrated that the methanol extract of the petiole of *E. crassipes* had anticancer properties against the SK-Mel-5 cell lines. Many previous studies, such as those using different solvent extracts of roots, shoots, and leaves of *E. crassipes*, have validated this phenomenon [1,3,4,7,10,16]. The drug-like characteristics conclusively prove that the intrinsic compounds from *E. crassipes* have the potential to be a reliable source of possible anticancer pharmaceutical options in the future. It was also documented that the isooctyl phthalate from the plant extract had favorable action against cervical and hepatocellular cancer [15]. Phytol, a diterpene molecule from the leaf of *E. crassipes*, has been shown to have antibacterial, anti-inflammatory, diuretic, and anticancer properties [15,19].

When Tyagi and Agarwal analyzed the ethanolic extracts of roots, shoots, and leaves of *E. crassipes* using gas chromatography-mass spectrometry [19], they found secondary metabolites having anticarcinogenic, antibacterial, antioxidant, antidandruff, and antiproliferative activities. According to Khalid et al., stem and leaf extracts contain more effective antimicrobial compounds than root extracts [20]. Additionally, ethyl acetate extracts of leaves and stems of *E. crassipes* have also been shown to have minimal antimicrobial properties against *Salmonella typhi* and *Staphylococcus aureus* [21]. A recent study confirmed that the ethanol fractionate of leaves and shoots of *E. crassipes* was effective in killing both the larval and pupal stages of the filarial vector, *Culex quinquefasciatus* [22]. The leaf and shoot components of ethanol extract have a substantial anti-inflammatory action to reduce pain, which has also been documented in a prior study [23].

According to research by Lenora et al., *E. crassipes* is a reliable and sustainable alternative source of shikimic acid. Shikimic acid is a key intermediate in the metabolism of aromatic amino acids. Shikimic acid is an essential component in plants and microbes but is not produced by humans. It is the foundation of the antiviral medicine oseltamivir, which is very effective against the swine-origin hemagglutinin and neuraminidase (H1N1) human influenza virus, as well as seasonal influenza A and B viruses and avian influenza H5N1. Shikimic acid (3.25%) was detected in *E. crassipes* aerial parts [14]. Petioles showed the greatest levels of radical scavenging activity, with an IC_50_ of 6.411 ± 0.46 mg/ml, compared to gallic acid's IC_50_ of 0.516 ± 0.22 mg/ml, as reported in a systematic study by Bakrim et al. The highest degree of inhibition reported in this investigation against 2,2-diphenyl-1-picrylhydrazyl (DPPH) radicals was achieved by the methanolic extract of *E. crassipes* at 250 g/ml [8].

*Eichhornia crassipes* was presented as an ornamental plant to beautify the water bodies. It is an invasive weed that can swiftly attain densities of more than 60 kg/m^2^. As a result, it substantially clogs the water bodies, causing a wide range of risks varying from environmental, economic, and societal fallout [24]. It threatens biodiversity, causes eutrophication, provides habitat for parasites, clogs fresh streams, has an impact on farming and fisheries, and impedes maritime transportation. Existing control strategies have proven ineffective in halting its exponential proliferation. Henceforth, *E. crassipes* have been extensively studied in recent decades due to their influence on ecosystems. However, its eradication will necessitate a substantial investment [19,25]. Water hyacinth reduces the ecological integrity of the environment it inhabits by obscuring sunshine. This decreases the growth of phytoplanktons, as well as other macrophytes, which in turn lowers the productivity of other living creatures in the water bodies [26].

Reactive oxygen species (also known as free radicals) are any oxygen-containing molecules that are highly reactive and may be produced by biological or physiological systems, environmental pollution, or other endogenous causes [17,20]. Mitochondrial dysfunction may be caused by free radicals interacting with membrane lipids, nucleic acids, proteins, and enzymes. Vitamins, terpenoids, phenolic acids, lignins, stilbenes, tannins, flavonoids, quinones, coumarins, alkaloids, amines, betalains, and phenolic coumarins are only some of the antioxidant-rich metabolites found in plants. Researchers Jayanthi and Lalitha found that certain solvent extractions of *E. crassipes* had higher reducing power than the common antioxidant L-ascorbic acid [27], suggesting the potential for the development of useful antioxidant molecules. Thus, the presence of phytochemicals in this terrible aquatic weed, as well as considerable antioxidant and antibacterial activities, makes it a desirable plant to produce future antimicrobial prescription drugs [20,21].

It has been shown that crude *E. crassipes* extracts may yield therapeutically promising results throughout a broad polarity spectrum. Several types of cancer cell lines (including T47D, PC3, NCI-H322, and A549), as well as cervical cancer (HeLa), and tumors in mice have been studied using aqueous plant leaf fractions, suggesting that polar chemicals contained in plants may be used as therapeutic approaches. The efficacy of hexane-ethyl acetate fractions against HepG2 liver cancer, HeLa cervical cancer, Michigan Cancer Foundation-7 (MCF-7) breast cancer, and esophageal adenoid cystic carcinoma (EACC) cell lines demonstrates the potential of non-polar phytoconstituents as an anticancer treatment option [15]. Jayanthi and Lalitha have shown that *E. crassipes* preparations rich in antioxidants such as glutathione, ascorbic acid, and polyphenols may delay the onset of aging [27]. Swiss albino mice were used in acute oral toxicological studies using ethyl acetate extract, aqueous extract, and methanol fractionate of aqueous extract, and all three extracts were shown to be nontoxic up to 2000 mg/kg body weight [28]. Methanol extract from the plants was also found to possess anti-inflammatory and anti-ulcerogenic properties in rats [7]. In addition, *E. crassipes* extracts dissolved in 50% methanol showed promising anticancer efficacy against malignant melanoma cells in mouse models at a range of doses [29].

Mtewa et al. documented that methanol was utilized in the early phases of extraction because it can extract a wide spectrum of chemicals with varying polarities. Recently, the leaves and roots of *E. crassipes* were isolated for their benzene-1,4-diol (0.003%) and nonanedioic acid (0.002%). Both compounds were elucidated using a blend of spectroscopic approaches, and the postulated structures were validated using liquid chromatography-mass spectrometry [30]. The drug-like effects of the chemicals isolated from roots, leaves, petioles, flowers, and stems of *E. crassipes* are yet to be investigated in depth. It is essential to investigate these compounds from this intriguing plant further to develop superior anticancer therapeutics through clinical trials. However, a single experiment is inadequate to understand the various mechanisms involved in exploring the antioxidant effects of the phytoconstituents [7,15].

Professional utilization of water hyacinth could be an alternative for weed management in the near future, contributing to addressing the environmental and economic challenges faced by it. The presence of the phytomolecules in the vegetation indicates the necessity for extensive and comprehensive chemical, pharmacological, and biological investigations for optimization. Moreover, the development of these phytomolecules as prospective breakthroughs in pharmaceuticals and/or nutritional supplements may also be warranted. Therefore, *E. crassipes* should no longer be seen as only a noxious plant but rather as a resource base that, under the right management, may aid in the improvement of the community by way of healthcare administration [30].

## Conclusions

The current in vitro study suggests that the methanol extract of the petiole of *E. crassipes* has a modest antiproliferative action against the SK-Mel-5 cell line, a typical human melanocyte tumor cell line. When the concentration of the extract was raised, there was also an increase in cell growth inhibition. The study findings shed light on the anticancer activity of *E. crassipes*, making it an appropriate source of drug-lead chemicals for the development of innovative, safer, and cost-effective remedies for cutaneous ailments varying from rashes to awful melanoma.
